# Competitive advantage of a dengue 4 virus when co-infecting the mosquito *Aedes aegypti* with a dengue 1 virus

**DOI:** 10.1186/s12879-016-1666-0

**Published:** 2016-07-08

**Authors:** Marie Vazeille, Pascal Gaborit, Laurence Mousson, Romain Girod, Anna-Bella Failloux

**Affiliations:** Institut Pasteur, Department of Virology, Arboviruses and Insect Vectors, 25 rue du Dr Roux, 75724 Paris Cedex 15, France; Medical Entomology Unit, Institut Pasteur of French Guiana, Cayenne, French Guiana

**Keywords:** Dengue, *Aedes aegypti*, Co-infection, Competitive interactions

## Abstract

**Background:**

Dengue viruses (DENV) are comprised in four related serotypes (DENV-1 to 4) and are critically important arboviral pathogens affecting human populations in the tropics. South American countries have seen the reemergence of DENV since the 1970’s associated with the progressive re-infestation by the mosquito vector, *Aedes aegypti*. In French Guiana, DENV is now endemic with the co-circulation of different serotypes resulting in viral epidemics. Between 2009 and 2010, a predominant serotype change occurred from DENV-1 to DENV-4 suggesting a competitive displacement. The aim of the present study was to evaluate the potential role of the mosquito in the selection of the new epidemic serotype.

**Methods:**

To test this hypothesis of competitive displacement of one serotype by another in the mosquito vector, we performed mono- and co-infections of local *Ae. aegypti* collected during the inter-epidemic period with both viral autochthonous epidemic serotypes and compared infection, dissemination and transmission rates. We performed oral artificial infections of F1 populations in BSL-3 conditions and analyzed infection, dissemination and transmission rates.

**Results:**

When two populations of *Ae. aegypti* from French Guiana were infected with either serotype, no significant differences in dissemination and transmission were observed between DENV-1 and DENV-4. However, in co-infection experiments, a strong competitive advantage for DENV-4 was seen at the midgut level leading to a much higher dissemination of this serotype. Furthermore only DENV-4 was present in *Ae. aegypti* saliva and therefore able to be transmitted.

**Conclusions:**

In an endemic context, mosquito vectors may be infected by several DENV serotypes. Our results suggest a possible competition between serotypes at the midgut level in co-infected mosquitoes leading to a drastically different transmission potential and, in this case, favoring the competitive displacement of DENV-1 by DENV-4. This phenomenon was observed despite a similar replicative fitness in mono-infections conditions.

## Background

Dengue viruses (DENV) are critically important arboviral pathogens responsible for an estimated 50–100 million cases of dengue (DEN) and 500,000 cases of the more severe and sometimes fatal dengue hemorrhagic fever/shock syndrome (DHF/DSS syndromes) annually (reviewed in [1]). DENV is a single-stranded positive sense RNA virus belonging to the genus *Flavivirus* (family *Flaviviridae*). Each of four related serotype (DENV-1 to 4) is anti-genetically distinct, comprised multiple lineages [2] and requires mosquitoes to complete their transmission to a vertebrate host. Although improved clinical care has reduced case fatality rates, there are currently no specific antiviral therapies or commercial vaccines.

In endemic regions where all four DENV serotypes are present, varying predominance of each serotype has been observed from year to year. The development of air travels has facilitated rapid displacements of viremic patients and has led to the introduction of multiple DENV into new endemic locations where increased disease severity has been linked to the displacement and extinction of local lineages [3–7]. Extinction and emergence of new virus lineages can be attributed to stochastic events associated with genetic bottlenecks in the viral population size [4, 5, 8, 9]. Bottlenecks may be caused by spatio-temporal reduction in vector population sizes and/or as a consequence of increase in human herd immunity [7]. Alternatively, predominant lineage change can be also attributed to differential fitness for transmission as was evidenced firstly by the higher susceptibility of *Aedes aegypti* for the invading Asian DENV genotypes than for the American genotype it has displaced in some locations [10]. Other examples of predominant lineage change events have been documented at more regional scales [5, 7, 9, 11–14]. Predominant lineage change can be attributed to deleterious mutations which lead to a progressive elimination of a lineage by purifying selection [8, 12, 15, 16]. Moreover, other factors related to the vectors can be implicated; some genotypes appear to be less well transmitted by local vector species [10, 13, 17–20]. Replication efficiency of certain strains in the human host may induce higher viremias which in turn, better infect mosquitoes [21, 22]. Additionally, the outcome of infection can depend on the specific pairing of vector and pathogen genotypes through genotype-by-genotype (G x G) interactions [23].

Following World War II, no dengue outbreaks were reported in the Americas while epidemics repeatedly struck countries in Southeast Asia. This reduction was mainly due to the eradication program initiated by the Pan American Health Organization (PAHO) to control the mosquito *Ae. aegypti*. Disruption of the program in the early 1970s however allowed a progressive reinfestation by *Ae. aegypti* [24]. In French Guiana, DEN became endemic with epidemics occurring every 4–6 years [25] and the first cases of DHF being reported during the 1991–92 DENV-2 outbreak [26]. While these outbreaks often coincided with the co-circulation of different serotypes [27], a predominant serotype change occurred from DENV-1 to DENV-4 between 2009 and 2010 [28] suggesting a competitive displacement, a phenomenon commonly observed among DENV genotypes [29] and serotypes [5, 7].

While competitive displacement among DENV strains has traditionally been examined through the ability of newly introduced strains to better replicate in human host, replication efficiency in the mosquito vector has remained poorly understood [10, 19, 30, 31]. Co-infection of vectors by multiple DENV strains occurs in nature and has been well documented in *Ae. aegypti* populations from both Thailand [32] and Brazil [33], occurring either when the mosquito takes several blood-meals from hosts infected with different serotypes, or when it takes a blood-meal from a single co-infected host [34–37].

Here, we have addressed the question of whether a newly introduced DENV-4 strain can successfully out-compete a well-established DENV-1 strain during co-infection in two populations of *Ae. aegypti* collected in French Guiana using *in vivo* experimental infections. We examined the ability of both DENV strains to overcome the different physical barriers (midgut and salivary glands) and subsequently, be transmitted successfully through saliva.

## Methods

### Viruses and cells

DENV-1 and DENV-4 isolates were provided by the French National Reference Center for Arboviruses (Institut Pasteur of French Guiana); viruses were isolated from sera collected in 2009 from patients living in the city of Cayenne by two successive passages in mosquitoes by intrathoracic inoculation. After a 14-days period of incubation at 28 °C, mosquitoes inoculated with the sera were triturated in heated (56 °C for 30 min) FCS (Fetal Calf Serum) and the resulting supernatant used again for a second mosquito passage. The final supernatant was then passaged on *Ae. albopictus* C6/36 cells for production of the viral stocks.

*Aedes albopictus* cells (C6/36) were maintained in Leibowitz L-15 medium with 1 % non-essential amino-acids (Invitrogen), 10 % fetal bovine serum (FBS) and 1 % penicillin/streptomycin (P/S) (Invitrogen).

### Mosquitoes

Mosquito samples were fortuitously collected in December 2009 during the inter-epidemic period (after the DENV-1 and before DENV-4 outbreaks) in the city of Cayenne, French Guiana: one in an environment of dense housing “Center” (CEN) and one in an environment of scattered housing, “Madeleine” (MAD). The samples collected as larvae and pupae were brought back to the laboratory and reared until the adult stage (F0 generation) at 28 ± 1 °C with 80 % relative humidity and a 16 h:8 h photoperiod. *Ae. aegypti* adults were sorted, fed with 10 % sucrose solution, and blood-fed every two days to obtain eggs (F1 generation). Egg batches were then sent to the Institut Pasteur in Paris where larvae were reared to adult stage under standardized conditions (temperature: 26 °C ± 2 °C, relative humidity: 80 % ± 10 %, photoperiod: 12 h:12 h) in pans with tap water and yeast tablets. F1 females were used for experimental oral infections under BSL-3 conditions.

The Paea strain of *Ae. aegypti* provided by the Institut Louis Malardé (Tahiti, French Polynesia) and reared in Paris since 1994, was used for intrathoracic inoculation to isolate and amplify DENV strains.

### Mosquito experimental infection and viral dissemination

Infection assays were performed with 7 day-old females which were allowed to feed for 15 min through a chicken skin membrane covering the base of a glass feeder containing the blood-virus mixture maintained at 37 °C. The infectious blood-meal was composed of a virus suspension diluted (1:3) in washed rabbit erythrocytes isolated from arterial blood collected 24 h before the infectious blood-meal. To attract females to the glass feeder, ATP was added to the blood-meal at a final concentration of 5x10^−3^ M. Completely engorged females were kept in small cardboard containers and maintained with 10 % sucrose at 28 ± 1 °C for 14–21 days. Titers of the blood-meal were 10^5^ or 10^6^ ffu/mL for mono-infected meals and 10^6^ ffu/mL for each virus in co-infected meals.

For mosquitoes fed with a single serotype, dissemination status was determined *via* indirect immunofluorescent assay (IFA) on head squashes [38]. Dissemination efficiency represents the proportion of mosquitoes where the virus has successfully penetrated the midgut and subsequently colonized secondary organs and tissues (i.e., positive by IFA on head squashes). Mosquitoes inoculated with DENV-1 or DENV-4 were used as positive controls. Negative controls were mosquitoes inoculated with cell culture media.

For mosquitoes fed with both DENV serotypes, the infection, dissemination and transmission status were determined by qRT-PCR using primers specific to the capsid gene: D1-Lm/C/153/+ (GAG AAA CCG CGT GTC AAC TG) and TS2-Lm/C/219/- (GGA AAC GAA GGA ATG CCA CC). A standard curve was generated using duplicates from 10^2^ to 10^8^ copies of RNA synthetic transcripts per reaction [39]. Infection efficiency corresponds to the proportion of mosquitoes with an infected abdomen (thus an infected midgut). Sensitivity of the qRT-PCR for both serotypes was asserted by analyzing mosquitoes infected intra-thoracically by one serotype.

### Transmission assessed with saliva collection and titration

At 14 or 21 days post-infection (dpi), surviving mosquitoes were chilled, and their wings and legs removed. Proboscis was inserted into a filter tip containing 5 μL FCS for 45 min. Serum containing the saliva was expelled under pressure into a 1.5 mL tube containing 45 μL Leibovitz L15 medium supplemented with 10 % FCS. For mono-infected mosquitoes, 40 μL of this sample were added to monolayers of C6/36 cells in 96-well plates. Infectious particles were detected by the foci forming technique using an immunofluorescent assay.

DENV-infected cells were incubated 5 days at 28 °C under an overlay consisting of 50 % of Leibovitz L-15 medium supplemented with 5 % FCS and 50 % of carboxyl methyl cellulose (CMC). Cells were fixed with 3.6 % formaldehyde at room temperature (RT) for 20 min and foci were detected by IFA.

For mosquitoes co-infected with the two serotypes of DENV, saliva was collected at 21 dpi to enhance the transmission rate and the whole sample (50 μL) was added to a monolayer of C6/36 cells in a 96-well plaque. After an incubation of 6 days, the supernatant was collected for RNA extraction (Nucleospin Tissue XS Macherey-Nagel) and analyzed by qRT-PCR to quantify RNA from each serotype. The cells were then fixed with 3.6 % formaldehyde and treated as described above to assert infectivity.

### Immunofluorescence assay

After a first incubation of 4 min with PBS + 0.5 % Triton X-100 (Sigma) at RT and three washes in PBS, cells were incubated 120 min at RT with a mouse ascitic fluid (provided by the French National Reference Center for Arbovirus, Institut Pasteur Paris) at a dilution of 1:100. After three washes with PBS, cells were incubated 60 min at RT with fluoresceine-conjugated goat anti-mouse IgG antibody (Biorad) at a 1:80 dilution in PBS 1X. After a final wash in PBS, foci were counted under a fluorescent light. The titer of infectious particles per saliva was expressed as ffu/saliva. Transmission rate corresponds to the proportion of mosquitoes with infectious saliva among mosquitoes able to disseminate the virus beyond the midgut barrier.

### Statistical analysis

To detect significant differences in infection, dissemination efficiencies and transmission rates among experimental groups, comparisons were done using the Fisher’s exact test (Stata software, StataCorp LP, Texas, and USA).

## Results

### Mono-infections of mosquitoes

**Fig. 1:**
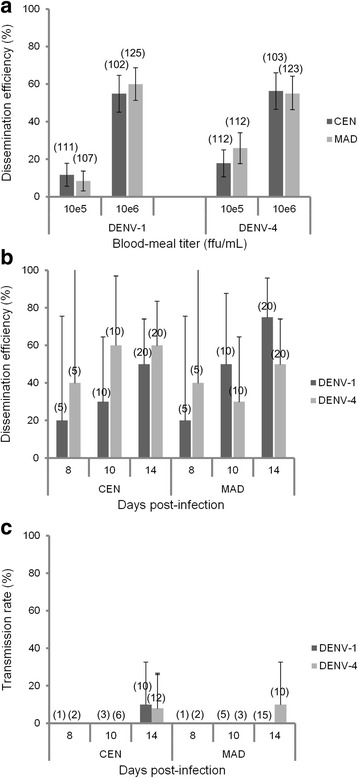
Mono infections of *Aedes aegypti*. Mono-infections with DENV-1 or DENV-4 of two populations of *Aedes aegypti* (CEN and MAD) collected in French Guiana in 2009. (**a**) Dissemination efficiency 14 days after DENV-infected blood-meals provided at a titer of 10^5^ or 10^6^ ffu/mL. (**b**) Dissemination efficiency and (**c**) transmission rate at different days after oral infection with DENV-1 or DENV-4 provided at 10^6^ ffu/mL. Error bars show confidence intervals (95 %). In brackets, the number of mosquitoes tested

To test whether competitive displacement of DENV-1 by DENV-4 can be attributed to variations in vector competence, we evaluated the ability of both DENV serotypes to be transmitted by *Ae. aegypti* in mono-infected blood-meals. The presence of virus in the head and in the saliva was used to measure viral dissemination and transmission, respectively. We orally infected two populations of *Ae. aegypti* (CEN and MAD) with either 10^5^ or 10^6^ ffu/mL of each serotype and determined viral dissemination rates at 14 dpi (Fig. 1a). Dissemination efficiencies increased significantly with the blood-meal titer (*p* < 0.05) and remained roughly similar regardless of virus (DENV-1, DENV-4) or mosquito population (CEN, MAD) (*p* > 0.05).

We next examined viral dissemination and transmission at 8, 10 and 14 dpi with a blood-meal provided at a titer of 10^6^ ffu/mL (Fig. 1b, 1c). Dissemination efficiencies were similar for both mosquito populations in all cases (by varying the day pi and the DENV) (*p* > 0.05). Assessment of transmission ability showed that less than 10 % of mosquitoes with disseminated infection were able to transmit DENV at 14 dpi (Fig. 1c). These results suggest that the extrinsic incubation period was between 10 and 14 dpi regardless of mosquito population or DENV serotype.

These findings suggest that there are no significant differences in dissemination or transmission rates between mosquito populations mono-infected with either DENV-1 or DENV-4.

### Co-infections of mosquitoes

**Fig. 2:**
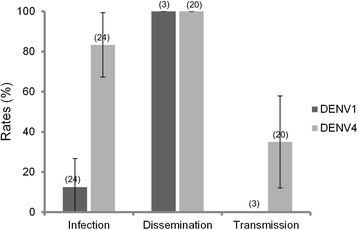
Coinfection of *Aedes aegypti*. Infection, dissemination and transmission rates in *Ae. aegypti* CEN population, 21 days after oral exposure to co-infected blood-meal DENV-1/DENV-4 provided at 10^6^ ffu/mL for each virus. Viral RNA was detected by qRT-PCR. Error bars show confidence intervals (95 %). In brackets, the number of mosquitoes tested

To determine if co-infection with two different DENV serotypes can affect vector competence, we analyzed viral infection, dissemination and transmission rates at 21 dpi, in co-infected *Ae. aegypti* CEN (Fig. 2). For each mosquito, saliva was collected then body (for infectious status) and head (for dissemination status) processed. Among 24 mosquitoes co-infected with both viruses, we found that at 21 dpi, more mosquitoes were infected at DENV-4 (83.3 % ± 16.1) than with DENV-1 (12.5 % ± 14.4) (*p* < 0.05). Mosquitoes detected positive for DENV-1 were all also positive for DENV-4. When examining the proportion of mosquitoes able to disseminate the virus beyond the midgut barrier after infection, dissemination was complete and similar for both DENV serotypes (*p* > 0.05): 100 % when examining DENV-1 (3 mosquitoes ensuring viral dissemination among 3 infected) and DENV-4 (20 mosquitoes ensuring viral dissemination among 20 infected). The 3 mosquitoes having disseminated DENV-1 had also disseminate DENV-4. Then, when analysing the transmission by detecting the virus in saliva of mosquitoes able to disseminate efficiently the virus, we showed that DENV-4 was detected in 35 % (±10.9) of mosquitoes (7 mosquitoes able to transmit among 20 able to disseminate DENV-4). However, DENV-1 was not recovered from any mosquito saliva. In brief, from 24 F1 mosquitoes having received a co-infected blood-meal with DENV-1 and DENV-4, 7 were able to transmit DENV-4 and none DENV-1. Taken together, these findings show that DENV-4 possesses a strong competitive advantage in co-infected mosquitoes at late dpi.

## Discussion

In situations where multiple DENV serotypes co-circulate, viral competition is most likely to occur in vectors where infection persists for life. In comparison, infection tends to be transient in vertebrate hosts, and is typically cleared by the host immune response. In French Guiana, DENV-4 has progressively displaced DENV-1 during the inter-epidemic period in 2009–2010. Using field-collected mosquitoes and low-passaged DENV, we showed that when mosquitoes were infected with either DENV-1 or DENV-4, no significant difference was observed in infection, dissemination or transmission rates. However, co-infection of mosquitoes with both the native DENV-1 and the newly introduced DENV-4 showed that DENV-4 possessed a strong fitness advantage compared to DENV-1.

In our experimental protocol based on co-infections with DENV-1 and DENV-4 provided at equal titers, the number of mosquito midguts infected with DENV-4 at 21 dpi was higher that the number of mosquito midguts infected with DENV-1. After having crossed the midgut, both DENV presented similar abilities to infect secondary internal organs. Critically, only DENV-4 was detected in saliva, confirming the selective advantage of DENV-4 during co-infection. During mono-infections in mosquitoes, the advantage of DENV-4 over DENV-1 was not observed.

Co-infections can lead to unique interactions requiring all aspects of the replication machinery. Examples include production of recombinant viral particles which may escape detection. This phenomenon has been previously reported within DENV serotypes, but appears to be a rare event [40–44]. Another potential interaction is that entire sections of the viral genome can be deleted followed by the replication of the remaining partial genome *via* complementation during co-infection with another functional virus. These defective interfering (DI) viral particles containing large genomic deletions may reduce the yield of fully functional DENV virions [45]. Once dissemination is completed, the virus reaches the salivary glands before being excreted in mosquito saliva. Only DENV-4 was detected in mosquito saliva at a proportion of 35 %. These results underline the importance of the midgut and the salivary glands where co-infection with both viruses has favoured DENV-4.

Co-infection of *Ae. aegypti* by different DENV serotypes can be described in nature, e.g. in Thailand [32], Brazil [33]. It can occur by feeding on a patient co-infected by two different serotypes [32, 34, 36, 37, 46, 47]. Our results show that in co-infected conditions, DENV-4 exhibited better transmission rates in mosquitoes and provide a potential explanation for the competitive displacement of DENV-1 by DENV-4 which occurred between 2009 and 2010 in French Guiana [28]. This finding is quite similar to the one observed in humans with live-attenuated tetravalent vaccine where replication of individual serotypes appears to be sensitive to the identity and concentration of co-infecting serotypes [48]. If one serotype is more likely to divert the replication machinery to its advantage in a co-infected mammalian cell, then more viral particles of this serotype and consequently, more neutralizing antibodies would be produced. This unbalanced immune response in hosts may result in an ineffective vaccine. Therefore, design of tetravalent vaccine should take into account interactions between serotypes.

Finally, viral displacements due to emerging epidemic events are often described; they should be closely studied using mixed infections models and not only single-strain infections that cannot reflect the outcomes of *in vivo* viral competition.

## Conclusions

We provided evidence of a competitive advantage of a DENV serotype on another using mosquitoes from the field and two autochthonous epidemic viral strains. This finding, along with other factors such as herd immunity in the human population, could have contributed to the switch from DENV-1 to DENV-4 in 2009–2010 in French Guiana. Other emerging events involving viral displacement should be carefully studied to estimate the frequency of such events.

## Abbreviations

ATP, adenosine triphosphate; CMC, carboxyl methyl cellulose; DEN, dengue; DENV, dengue virus; DENV-1, dengue serotype 1; DENV-2, dengue serotype 2; DENV-4: dengue serotype 4; DHF, dengue hemorrhagic fever; dpi, day post-infection; DSS, dengue shock syndrome; FBS, fetal bovine serum; FCS, fetal calf serum; ffu, fluorescent focus forming units; IFA, indirect immunofluorescent assay; P/S, penicillin/streptomycin; PAHO, Pan American Health Organization; PBS, phosphate-buffered saline; qRT-PCR: quantitative Reverse transcription polymerase chain reaction; RNA, ribonucleic acid; RT, room temperature
